# Commentary: The Hidden Effects of Dairy Farming on Public and Environmental Health in the Netherlands, India, Ethiopia, and Uganda, Considering the Use of Antibiotics and Other Agrochemicals

**DOI:** 10.3389/fpubh.2016.00288

**Published:** 2017-01-09

**Authors:** Habtamu Lemma Didanna

**Affiliations:** ^1^Wolaita Sodo Univeristy, Sodo, Ethiopia

**Keywords:** smallholder dairying, milk quality, breeding, Ethiopia

Recently, I read the abovementioned article that was published in *Frontiers in Public Health* 4(12) on February 24, 2016. I need to put my expert opinion on it especially in the Ethiopian context.

The original article on “The Application of Natural Livestock Approach” at this time to Ethiopian dairying system is inappropriate. It failed to address the function of dairy farming in satisfying the need of increasing demand of food/nutrition and livelihood security. The article also did not address options for meeting/minimizing the challenges of intensifying dairying on which the western/developed countries have ample experiences including maintaining milk safety, disease prevention/control, and manure management.

Hereunder, I want to explain briefly about smallholder dairying, which contributes great share to the dairy sector, though with little institutional support. I also argued against the authors’ approaches especially using only indigenous breeds and payment for residue-free milk currently.

## Smallholder Dairying

Smallholder dairying is a cost-effective and key source of nutrition and income to 300 million farm families globally (1, 2), plays an important social role in developing countries including Ethiopia, and is considered an important means of alleviating poverty (3, 4). A number of market-oriented/moderately intensive smallholder dairy farming has appeared mainly in the urban and peri-urban areas of the Addis Ababa and most regional towns and districts, which is a major milk supplier to urban setting.

## Milk Safety

In the formal milk market, producers supply milk to dairy cooperatives and/or private processing plants, which often implement quality control measures through two different quality tests: a lactometer reading and an alcohol test. They also pasteurize milk collected from smallholder dairy producers. However, there is lack of capacity of smallholder dairy producers in maintaining milk safety that can be managed through supporting the producers. According to Steen and Maijersb (5), the case study of Hiruth milk production and processing enterprise in Ethiopia indicated how milk processor’s effort tackles some of the problems in improving milk quality. Hiruth provides input such as feed to her suppliers on credit basis, and she also educates producers. Hiruth managed to build her supply base loyalty by assuring purchase and quality-based price premium (bacterial count and fat). This progressive incentive mechanism motivates milk producers to focus on quality and carry out investments such as the purchase of better quality feed, lower adulteration, and better storage conditions for the milk. Similarly, other stakeholders in the dairy value chain need to discharge their potential roles and responsibility to bring about sustainable smallholder dairying.

The challenge facing policy makers is to balance the objectives of consumer protection and dairy producers’ livelihood security. Addressing and managing food safety risks in milk and dairy products should involve all stakeholders across public and private sectors. Bringing about change and adoption of better practices for food safety should take into account a range of socioeconomic factors, the structure, size, and organization of the milk production and processing sector, and the scientific knowledge of producing a safe product. In this regard, a comprehensive risk-based preventive approach is required, from farm level through to processing, marketing, and consumption; up-to-date information systems are needed to provide relevant data on national dairy industries and information on available technology and training (6). FAO has a unique global perspective on this and needs to enhance its leadership and expand its capacity in providing both relevant technical information and strategic/policy advice to government and the private sector on sustainable dairy industry development (7).

In Ethiopia, therefore, all dairy stakeholders need to work in carefully planned and integrated manner to prevent and control milk quality/safety problems. The Ethiopian Meat and Dairy Industry Institute in collaboration with Livestock State Ministry is the most appropriate body to coordinate/organize those stakeholders. The regulatory issue can be handled by Veterinary and Feed Administration and Control Authority and Food, Medicine, Health Care and Administration Authority. Before putting enforcement of the quality standards, there is a need to provide land security/dairy parks, input supply, and support services (feeding, breeding, health care, marketing, barn conditions, and manure management) for peri/urban smallholder dairy producers (youth, women, elderly people, etc.) in the pilot period in which the roles of the stakeholders are also tested before the actual implementation of the quality control measures.

## Ethiopian Cattle Breeding Strategy

One of the strategies to meet the increasing milk demand is through production of genetically improved breeding stock plus management/feeding and health care/practices. In this regard, the potential option in Ethiopia is crossbreeding indigenous cows with exotic dairy breeds. This is evidenced by Norman and Defilcho (8) who stressed that indigenous cattle in the tropics (*Bos indicus*) are well adapted to the local conditions, but their milk production potential is less than the *Bos taurus* in temperate regions. Therefore, in the tropics, dairying on the basis of indigenous cattle alone would not be a suitable option to meet the increasing demand for milk and milk products. The most favored alternative so far has been crossbreeding, to incorporate the hardiness of *B. indicus* types with the productive capacity of *B. taurus* animals.

Any cattle breeding strategies in the mixed crop-livestock system of Ethiopia should re-orient itself toward solving problems prevailing in the system on the one hand and market-oriented production on the other hand (9). Depending on the production system (mixed or peri/urban), exotic genes level could be at 50 or 62.5% (10). In addition, indigenous cattle breeds in the mixed, agropastoral, and pastoral areas need genetically characterized and conserved/used as they are valuable genetic resources in a changing climate (11). According to Central Statistical Agency (12), 98.59% of the total cattle in the country are local/indigenous breeds.

## Concluding Remarks

Farmers in resource-constrained countries traditionally use few external inputs, such as allopathic medicines and antibiotics, and follow grazing-based extensive or semi-intensive production systems. In many ways, they are thus closer to organic farming systems, though largely by default. However, these farmers convert into ecological intensification (i.e., sustainable farming) due to various reasons (13). Residue from most of smallholder dairy producers is very little; instead, chemical/pesticide use in crop and vegetable farming needs investigation for remedial action, probably through integrated pest management. If any, overuse and misuse of antibiotics by dairy farms can be reduced through effective veterinary services and withdrawal period. The problem mentioned in the original article (antibiotic use and loss of hardy breed at the expense of high yielding) is severe in more intensified/industrial dairy farming like the case in Europe, including The Netherlands.

Smallholder dairying is considered an important source of food/livelihood and nutrition security in Ethiopia. However, the challenges faced by smallholder dairy producers can be managed (14) through support services that enhance their contribution in sustainable ways. Hence, various stakeholders in the dairy value chain need to work closely. Dairy projects/interventions should also consider integrated use of both ethno-veterinary/herbal and veterinary medicine and capacity building, technical and material support on maintaining milk safety, controlled crossbreeding/AI and/or bull services, maintaining indigenous breeds, manure management, health management/diseases of intensification, simple milk record keeping, etc. In this regard, the experiences of successful smallholder dairy programs can be taken into consideration.

## Author Contributions

The author confirms being the sole contributor of this work and approved it for publication.

## Conflict of Interest Statement

The author declares that the research was conducted in the absence of any commercial or financial relationships that could be construed as a potential conflict of interest.

## References

[1] World Bank. Agricultural Investment Sourcebook. Washington, DC (2005).

[2] Ogola TDO Kosgey IS Breeding and development of dairy goats: Eastern Africa experience. Livest Res Rural Dev (2012) 24(1).

[3] Ahmed MAM Ehui S Assefa Y Dairy Development in Ethiopia. IPFRI-EPTD, Discussion Paper 123. Washington, DC: Environment and Production Technology Division (2004).

[4] Somda J Kamuanga M Tollens E Characteristics and economic viability of milk production in the smallholder farming systems in Gambia. Agric Syst (2005) 85:42–58. 10.1016/j.agsy.2004.07.011

[5] Steen M Maijersb W Inclusiveness of the small-holder farmer key success factors for ethiopian agribusiness development. Int Food Agribusiness Manage Assoc (2014) 17:83–8.

[6] FAO. Milk and Dairy Products, Post-Harvest Losses and Food Safety in Sub-Saharan Africa and the Near East. Rome (2005).

[7] FAO. Milk and Dairy Products in Human Nutrition. Rome (2013). Available from: www.fao.org/publications

[8] Norman GA Defilcho PE Effects of breed and nutrition on the productive traits of Zebu, Charolais and crossbreed beef cattle in South Eastern Brazil. Part 1. Body and gross carcass composition. Meat Sci (1980) 5:425–38. 10.1016/0309-1740(81)90041-3 22054603

[9] Efta K Dessie T Yilma Z Haile A Cattle breeding strategies for sustainable genetic improvement in Ethiopia. Proceedings of the 17th Annual Conference of ESAP Addis Ababa, Ethiopia (2009).

[10] Haile A Joshi BK Ayalew W Tegegne A Singh A. Genetic evaluation of Ethiopian Boran cattle and their crosses with Holstein Friesian in central Ethiopia: milk production traits. Animal (2009) 3(4):486–93. 10.1017/S1751731108003868 22444371

[11] Habtamu L Domestic animal biodiversity in Ethiopia and its threats and opportunities with emphasis to changing climate: an overview. Adv Life Sci Technol (2012) 6:33–9.

[12] Central Statistical Agency (CSA). Agricultural Sample Survey. Report on Livestock and Livestock Characteristics. Volume II. Statistical Bulletin 583. Addis Ababa: Central Statistical Agency (2016).

[13] Willer H Kilcher L, editors. The world of organic agriculture: statistics and emerging trends 2011. International Federation of Organic Agriculture Movements. Frick, Switzerland: Bonn & Research Institute of Organic Agriculture (2011).

[14] Chagunda MGG Mwangwela A Mumba C Dos Anjos F Kawonga BS Hopkins R Assessing and managing intensification in smallholder dairy systems for food and nutrition security in Sub-Saharan Africa. Reg Environ Change (2015) 16:2257 10.1007/s10113-015-0829-7

